# Molecular Mechanism of Arsenic-Induced Neurotoxicity including Neuronal Dysfunctions

**DOI:** 10.3390/ijms221810077

**Published:** 2021-09-17

**Authors:** Manisha Thakur, Mahesh Rachamalla, Som Niyogi, Ashok Kumar Datusalia, Swaran Jeet Singh Flora

**Affiliations:** 1 Department of Pharmacology and Toxicology, Transit Campus, National Institute of Pharmaceutical Education and Research-Raebareli, Lucknow 226002, India; manishathakur0298@gmail.com (M.T.); ashok.datusalia@niperraebareli.edu.in (A.K.D.); 2 Department of Biology, University of Saskatchewan, Saskatoon, SK S7N 5E2, Canada; mar935@mail.Usask.ca (M.R.); som.niyogi@usask.ca (S.N.); 3 Toxicology Centre, Department of Biology, University of Saskatchewan, Saskatoon, SK S7N 5E2, Canada

**Keywords:** arsenic, environmental toxicity, myelination, neurotoxicity

## Abstract

Arsenic is a key environmental toxicant having significant impacts on human health. Millions of people in developing countries such as Bangladesh, Mexico, Taiwan, and India are affected by arsenic contamination through groundwater. Environmental contamination of arsenic leads to leads to various types of cancers, coronary and neurological ailments in human. There are several sources of arsenic exposure such as drinking water, diet, wood preservatives, smoking, air and cosmetics, while, drinking water is the most explored route. Inorganic arsenic exhibits higher levels of toxicity compared its organic forms. Exposure to inorganic arsenic is known to cause major neurological effects such as cytotoxicity, chromosomal aberration, damage to cellular DNA and genotoxicity. On the other hand, long-term exposure to arsenic may cause neurobehavioral effects in the juvenile stage, which may have detrimental effects in the later stages of life. Thus, it is important to understand the toxicology and underlying molecular mechanism of arsenic which will help to mitigate its detrimental effects. The present review focuses on the epidemiology, and the toxic mechanisms responsible for arsenic induced neurobehavioral diseases, including strategies for its management from water, community and household premises. The review also provides a critical analysis of epigenetic and transgenerational modifications, mitochondrial oxidative stress, molecular mechanisms of arsenic-induced oxidative stress, and neuronal dysfunction.

## 1. Introduction

Arsenic is recognized as a primary environmental pollutant that has substantial health impacts on human and other species. It is also ranked first in the priority list of Agency for Toxic Substances and Disease Registry (ASTDR), USA till 2020 (https://www.atsdr.cdc.gov/spl/index.html, accessed on 17 July 2021) [1]. Arsenic is a ubiquitous environmental contaminant widely distributed in the surrounding environment. Further spread of Arsenic is promoted through anthropological actions including smelting, burning of fossil fuel, and use in pesticide production responsible for its increased levels in earth, water, air, agricultural and aquatic food [2,3]. Increased arsenic levels in the environment have thus become a serious human health concern is widely distributed globally [4]. Developing countries such as Bangladesh, India, Mexico, and Taiwan are highly impacted by arsenic contamination in groundwater [4,5,6]. Epidemiological studies suggest that arsenic and its related compounds are responsible for causing various types of cancers, coronary and neurological ailments [7]. Arsenic toxicity is influenced by its chemical speciation, as inorganic arsenic exhibits a higher level of toxicity compared to organo-arsenicals. Inorganic arsenic is a potent carcinogen and causes malignancies in lungs, kidneys, skin, urinary bladder, and liver [8]. Chronic arsenic exposure via drinking water is one of the major factors for the greater risk of noncancerous ailments such as pigmentation, hyperkeratosis, cardiovascular disorders, hypertension, neurological, liver and kidney disorders, and diabetes [9]. Increased arsenic in the environment also impacts the health of aquatic species [10]. Arsenic present in sediments is biologically available through diet to benthic fish [10]. Dissolved arsenic levels in aquatic ecosystems in many developing countries have been reported to be higher than the permissible limit (10 µg/L) set by World Health Organization (WHO). This might be responsible for the disturbed physiological functions such as ion regulation, gene expression, enzyme and immune functions, growth and repair of tissue matrix, reproduction, and development [10]. Several studies in rodents, fish and invertebrates suggest that increased arsenic accumulation may alter the normal physiological function of organisms by directly or indirectly promoting the initiation of disease [10,11,12,13,14]. Arsenic is a strong reducing agent and interacts with other molecules such as sulphur, chloride and oxygen. It’s interactions with carbon containing molecules results in the formation of organic arsenic [15]. In addition, binding of arsenic with certain metals and charged ions such as Ca or Mg promotes the adsorption of As(V) in the solid particulate phases [16]. In addition, the effects of arsenic and its critical interactions might acknowledge new platforms of recent understanding on the diverse activities. Thus, the complete understanding of pathological effects and molecular mechanism of arsenic are crucial to mitigate its harmful effects on various species health.

Briefly, frequent monitoring of arsenic levels and associated health effects in various organisms provides insights into the overall health and also acts as a guard for prospective effects on the food chain [17]. Studies have shown severe impacts of arsenic on learning, memory and cognitive deficiencies in animals, suggesting brain to be a vital target for arsenic-induced toxicity [18]. Various inorganic and organic arsenicals appeared to accumulate in different areas of brain [19]. Arsenic easily crosses blood–brain barrier which further potentiates its accumulation in different brain regions leading to various neurological disorders [20]. Arsenic causes detrimental impacts on morphology and physiological changes in the brain cells. The brain is susceptible to oxidative stress because of its high energy requirement, and arsenic exposure causes oxidative stress damage via reduced antioxidant enzymes in the brain [21]. In fact, increased oxidative stress is a major mechanism of arsenic-induced neurotoxicity [22]. In this review article, we focus on the various toxic mechanisms by which arsenic induces neurobehavioral diseases. In addition, we discuss recent advances in understanding arsenic neurotoxicity, including the role of epigenetic modulations, mitochondrial oxidative stress, and neuronal dysfunction.

## 2. Means of Human Arsenic Exposure

Arsenic is a metalloid, one of the most ubiquitous environmental pollutants, and occurs naturally in the groundwater level with reported cancerous and noncancerous health impacts [23,24]. Arsenic is highly toxic in its inorganic form, and widespread arsenic contamination of Bangladesh tube well water is an example of chronic inorganic arsenic exposure [25]. Chronic exposure to arsenic poisoning is also known as arsenicosis, while. skin abrasions and skin melanoma are the most characteristic consequences [26]. Most of the effects of chronic exposure depend on the level and duration of exposure and include peripheral neuropathy, gastrointestinal symptoms, diabetes, cardiovascular diseases and developmental toxicity. Organic arsenic forms that are present in seafood are less harmful compared to inorganic arsenic as they are rapidly eliminated from the body [27]. Thus, the abundant arsenic presence in the earth’s crust requires scientific communities to work on its regulation and safe management strategies. Some of these strategies are discussed below in brief.

### 2.1. Drinking Water

The major resource for drinking water is groundwater, and the elevated concentrations of arsenic in ground water has been correlated with numerous adverse human health impacts [28,29]. Initially, WHO recommended the maximum acceptable arsenic concentration in drinking water to be 50 µg/L, but later revised it to 10 µg/L up to 1992 [30,31]. Contamination of groundwater with inorganic arsenic is identified to be the primary route for human exposure, and eventually responsible for various health effects which were reported in Bangladesh, Vietnam, China, Taiwan, Argentina, and Canada [32,33,34,35,36,37,38].

### 2.2. Diet

Intake of arsenic through contaminated food is another major source of chronic arsenic exposure in humans, mediated by agricultural crops being cultivated using arsenic-contaminated groundwater. Previous research has shown that polluted groundwater used to grow crops for human consumption could be a major source of arsenic ingestion [39]. A similar study found that vegetables imported to the UK from Bangladesh had 2–100 fold higher arsenic concentrations than vegetables farmed in North America and UK, although arsenic species were not reported [40]. In another report, arsenobetaine and arsenosugars were detected in crustaceans and seaweed respectively [41].

### 2.3. Industrialized and Wood Preservatives

Arsenic is extensively used in the industry for production of antifungal wood preservatives, which can be the source of contamination for soil. Ignition of well-conserved woods (pressure treated with heavy metals) were discovered to be a source of arsenic contamination [42]. As stated by the ASTDR, even though application of arsenic-containing wood preservatives has been prohibited for several household uses, it is still being used in industries. The US Environmental Protection Agency (US-EPA) in 2008 drafted Reregistration Eligibility Decision Documents (REDs) to protect exposed workers from arsenic effects. Recently, the harmful effects of chromated arsenicals in response to public health were reviewed by the EPA and special attention was been given to rationalize its use in order to mitigate associated risks [43,44]. Arsenic is also used in pharmaceuticals and glass industries for the production of leather preservatives, pigments, antifouling paints, and poisonous baits. Some arsenic-containing composites are also utilized in the production of optics and microelectronics [45]. Sodium arsenite has frequently been applied as a herbicide and nonselective soil sterilant [46].

### 2.4. Smoking

The literature suggests that smokers have a decreased ability to methylate ingested arsenic, as smoking causes increased percentage MMA (mono-methyl arsenic) and lowers percentage DMA (dimethyl arsenic) and the secondary methylation index compared to nonsmokers [47]. It is reported that elevated urinary excretion of arsenic and MMA are found in smokers compared to nonsmokers [48]. In another study, arsenic exposure through cigarette smoking and drinking water resulted in a synergistic toxic effect [49]. Smoking has also been associated with a higher risk of bladder cancer. A study from the USA revealed that smokers exposed to elevated concentrations of arsenic in drinking water (200 µg/L), had a greater hazard of developing urothelial carcinoma than those who were exposed only through smoke [50,51].

### 2.5. Air

Arsenic in air mainly exists as particulate matter and is usually a combination of arsenite and arsenate, with a minor amount of organic arsenic. Methylated arsenic is present in minor amounts in air, whereas inorganic arsenic as trivalent and pentavalent arsenic are the major components [52]. Inhalation of over 10 ppm is lethal [53], though the exposure of arsenic through air is low at between 0.5 × 10^−3^ to 30 × 10^−3^ µg/m^3^ [54]. The US Environmental Protection Agency (EPA) has projected that around 40 × 10^−3^ to 90 × 10^−3^ µg of arsenic is inhaled by humans per day [55,56].

### 2.6. Cosmetics

Cosmetics are another potential source of arsenic exposure to humans. Assessment of dermal absorption of drugs depends on many factors, and currently there are no standards available for impurities testing. Few nations have determined the concentrations of toxic metal and metalloids, including arsenic, in cosmetic products [57,58]. The available information documents that several sources exist regarding the toxicity of metals through cosmetics. Lucia Atz et al. 2009 determined trace elements levels of cadmium, chromium, copper, and arsenic in cosmetic products like eye shadow and lipsticks using atomic absorption spectroscopy following an acid digestion method. They reported a maximum concentration of 11.1 mg As/kg arsenic in eye shadow [59,60].

## 3. Metabolic Pathway of Arsenic

**Figure 1:**
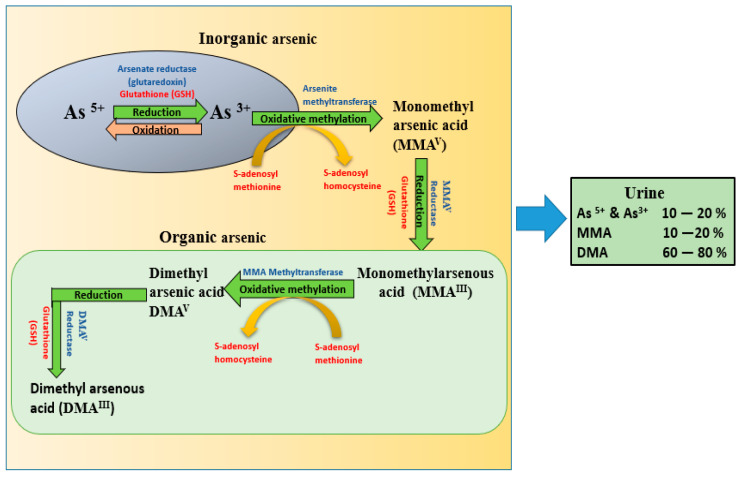
Metabolic pathway of inorganic arsenic demonstrating the reduction of arsenate to arsenite with the enzyme arsenate reductase mediated by glutathione (GSH), which further undergoes oxidative methylation through the enzyme arsenite methyltranferase mediated by S-adenosyl methionine, with conversion to MMA and DMA. Finally, all the metabolites are excreted through urine, among which DMA is the major metabolite (60–80%). (As- arsenic, DMA-dimethyl arsenic acid, MMA-monomethyl arsenic acid, GSH-glutathione).

Scientific studies indicate that inorganic arsenic methylation was incomplete, and urinary metabolite excretion varied from person to person, though arsenic exposure was same in all the populations [61]. Inorganic arsenic is associated with toxicity and is reduced from As(V) state to As(III) by arsenate reductase enzyme. The generated trivalent species are highly active and toxic [62]. Arsenic is primarily metabolized and detoxified through oxidative methylation in the liver in the presence of methyl donor S-adenosylmethionine (SAM). The co-factor used is glutathione (GSH) with arsenic methyltrasferase resulting in the production of monomethylarsonic acid and dimethyl arsenic acid which are finally excreted through urine (Figure 1) [63].

As discussed above, the toxic potential of arsenic primarily depends on the form of arsenic in the body. Arsenic initially absorbed through various routes enters the bloodstream and is taken up by red blood cells (RBC), white blood cells (WBC), and other cells. Arsenate, on the other hand, is reduced to arsenite and is subsequently methylated to monomethyl arsonate (MMA), and dimethyl arsenate (DMA) [64]. Reduction of arsenate to arsenite is essential before methylation and requires glutathione (GSH) and methyl group transferase S-adenosyl methionine [65,66]. It is important to note that the absorbed form of arsenic depends on the nature of arsenic because all the absorbed arsenic is not in the pentavalent form. The main urinary metabolite found in urinary arsenic is DMA (60–80%). Even though methylation is the most important pathway for arsenic detoxification, its effectiveness in humans seems to decline when exposed to higher doses (Figure 1).

## 4. Neuronal Effects of Arsenic

Neuronal alterations due to metals/metalloids are well documented. Arsenic exposure is known to cause various neurological disorders through diverse molecular mechanisms such as cytotoxicity, increased reactive oxygen species (ROS), chromosomal aberrations and cellular DNA damage. These genotoxic effects are the major cause of degenerative changes in neurological systems [67,68]. Various epidemiological studies have revealed a correlation between increased arsenic in drinking water and neurological behavioral disorders such as decreased locomotor activity, impaired cognitive functions, and prenatal complications. Arsenic easily crosses the blood-brain barrier and can accumulate in various parts of the brain, such as the striatum and hippocampus, which further potentiates arsenic toxicity and tissue injury [69,70].

There is much evidence for the neurological impacts of arsenic in animal models. However, very few epidemiological reports are available on the influence of arsenic on adult mental health and cognitive performance [71,72]. Epidemiological and toxicological studies indicate that arsenic is a developmental neurotoxicant and is responsible for inducing intellectual and cognitive disabilities in humans [73]. Studies conducted in Bangladesh, India, Mexico, and Taiwan have indicated that chronic exposure to even very low levels of arsenic (<10 µg/L) reduced IQ and memory performance in exposed children [72]. The experimental research carried on animals has expanded our understanding of the outcomes of new and potential neurotoxic components. Arsenic can induce neurotoxic effects by altering the levels of neurotransmitters such as serotonin, dopamine and norepinephrine in the brain [74,75]. The detrimental effects of arsenic are largely influenced in the developmental stages. Exposure to arsenic could perturbate neurological complications, severely affecting memory and learning, anxiety and mood instability [76,77]. Several mechanisms correlate arsenic toxicity with reduced synaptic signaling, plasticity and neurogenesis [78].

The literature includes possible mechanisms involved in the impaired cognitive performance in adults with arsenic exposure. Peripheral nerve neuropathy, altered sensory function and reduced conduction velocity were observed in humans who were subjected to elevated levels of inorganic arsenic [79,80]. Even a single dose of 50 ppb inorganic arsenic in water in adult mice led to peripheral neuropathy, which resulted in the reduction of motor conduction velocity and abnormalities in action potentials in sensory nerves in offspring [81]. It has also been observed that arsenic exposure caused loss of neurofilaments and decreased expression of fibroblast proteins in rat sciatic nerves [82]. Arsenic-induced oxidative stress and demyelination, and morphological impacts on peripheral neurons suggest that these impacts further impair the transmission of signal transduction from the peripheral nervous system to the CNS leading to detrimental impacts on mental health [80].

The mechanisms linking arsenic exposure to neurodegeneration are complex, and our understanding of these mechanisms is continuously evolving [83]. Previous studies have suggested that arsenic might cause neurodegeneration through various mechanisms, the most studied including oxidative stress, inflammation, and mitochondrial dysfunction [84,85]. In a case control study, increased urinary arsenic excretion in patients was observed in correlation with enhanced risk of progression of Alzheimer’s disease (AD). AD is a progressive neurological disorder characterized by the formation of neurofibrillary tangles and β-amyloid (Aβ) plaques [86]. Arsenic-induced dementia and vascular injury were also reported in in-vivo studies [87]. Chronic exposure to arsenic in rats caused behavioral deficits which were associated with high levels of amyloid-β, increased advanced glycation-end products and β-secretase (BACE-1) activity in the brain [83]. Arsenic exacerbated amyloid-β and phosphorylated tau in transgenic AD rodent models, which were mediated through bioenergetic disfunction and modified redox metabolism [83]. Interestingly, arsenic reportedly induced behavioral deficits and neurodegeneration via increased production of the Aβ_(1–42)_, amyloid precursor protein (APP) and BACE-1 [88]. These proamyloidogenic effects of arsenic were synergized when coexposed with other heavy metals, and these effects were mainly mediated by oxidative damage and neuroinflammation of brain tissues [89]. Arsenic increases proinflammatory cytokines levels in astrocytes, which are a subtype of glial cell in the central nervous system and mediate brain homeostasis and neuronal metabolism. Any imbalances/insults in glial cells lead to increased levels of amyloid precursor protein [90]. Arsenic toxicity may also synergize with DA (dopamine) to cause neurotoxicity, and cause α-synuclein aggregation, which is a hallmark of Parkinson’s disease [91].

Perinatal exposure of adult mice to 50 µg/L arsenic via drinking water was found to cause depression and depression-like behavior in the offspring [92]. The study also reported elevated serum corticosterone levels and subsequent reduction of the whole hippocampal corticotrophin-releasing factor (CRFR1), increased dorsal hippocampal serotonin 5HT_1A_ receptor binding and receptor-effector coupling. These observations imply that perinatal exposure to arsenic may interrupt the regulatory connections between the hypothalamic-pituitary-adrenal (HPA) axis and the serotonergic system in the dorsal hippocampus. These changes significantly induced depressive behavior in offspring [75,92]. Several rodent studies indicated that chronic exposure to low and moderate levels of arsenic could significantly alter the levels of NE, DA, and 5-HT in the brain; such effects have often been reported to occur in a sex-specific manner [70,71,72,74,75,79,80,83,89,91,93,94,95,96,97,98,99,100,101]. Arsenic toxicity is more common in males (53.7%) than females (46.3%). It also causes fatal effects on the male reproductive organs and development [102]. However, low levels of arsenic (~1.321 mg) affect pregnant females and their offspring. The newborns from the arsenic exposed females showed low socio economical communications and malnutrition with an effect on growth and development [103]. Collectively, this evidence suggests that pregnant women are at a higher risk to arsenic.

To date, the neurobehavioral implications of chronic arsenic exposure have not been investigated fully in any species. Dipp et al. [95], chronically exposed different life stages of zebrafish (larval, juvenile and adult) to waterborne arsenic (50–500 µg/L) and subsequently examined motor function, social and cognitive behaviors, and anxiety-like behaviors. They reported altered motor function in embryos and adults at 500 µg/L arsenic exposure, and an increase in anxiety behavior in juveniles and adults at the same exposure. Associative learning behaviors were also impacted at 500 µg/L exposure, but only in adults [95]. The major potential mechanism of arsenic neurotoxicity could be oxidative stress. When adult zebrafish were chronically exposed to arsenic trioxide (50 µg/L for 90 D), upregulation of catalase (Cat), glutathione peroxidase (Gpx1), copper/zinc superoxide dismutase (SOD1), and manganese superoxide dismutase (SOD2) were recorded in the brain. In addition, mitochondrial cytochrome c oxidase1 (Cox1) and B-cell lymphoma 2 (Bcl2) were also upregulated, indicating the initiation of apoptosis in brain cells [104]. Even low levels of arsenic at 10 µg/L could cause long-term memory loss in zebrafish when tested with a one-trial inhibitory avoidance test. This is a behavioral task aimed at evaluating learning and memory mechanisms currently available to zebrafish, and is associated with increased protein oxidation in the brain [105].

### 4.1. Neurotransmitter Mediated Impacts of Arsenic

There are several neurotransmitters responsible for the communication between cells within the brain. Arsenic has neurotoxic effects on these neurotransmitters and inducible effects on dopamine (DA) and serotonin (5-HT) levels due to regulation of norepinephrine (NE) levels [106]. Arsenic also alters the levels of GABA, glutamate and other biogenic amine levels, as well as biogenic amines (5-HT, NE and DA) and nitric oxide [107]. Nagaraja et al. [108] reported that inorganic arsenic consumption decreased acetylcholinesterase activity, which helps in metabolism of acetylcholine in rodents [108]. Poor outcomes in learning and memory could be mechanistically linked with altered levels of neurotransmitter release [109]. Other research groups have identified that the neurotoxic effects of arsenic are mediated via reduced glutamate levels and mGluR5 expression in the hippocampus [110]. In addition, exposure to arsenic in rats reduced the activity of acetylcholinesterase (ACHE) in the central compartments of the brain [111]. Similarly, other studies evaluated the reduced expression of homovanillic acid (HVA) and 3,4-dihydroxyphenylacetic acid (DOPAC) in mice treated with arsenic [112].

Another potential mechanism of arsenic-induced neurobehavioral alterations could be mediated by the transcriptional regulation of ectonucleotidases. The uncoupling of oxidative phosphorylation is linked with the formation of arsenate and ADP complexes [113]. Mitochondrial formation of ATP from ADP and PO4 provide cells with energy. The formation of ADP + arsenic can occur faster than ATP formation, thereby decoupling ATP production. Significant decrease in the mRNA expression of NTPDase members (entpd2_mg, entpd2_mq) and Ecto-5′-nucleotidase, eventually results in a reduction of ATP/ADP and AMP hydrolysis [114]. Arsenic-mediated alterations in the activities and mRNA levels of ectonucleotidases might be responsible for decreased adenosine levels, which could alter movement and anxiety reactions in zebrafish [114]. Arsenic could also act on the cholinergic system by interacting with thiol (-SH) groups involved in the uptake of choline and disulfide group of acetylcholinesterase [111,115,116]. Moreover, rodents exposed to arsenic showed a decrease in glutamic acid decarboxylase (GAD) expression in some areas of the brain, while glutamate (Glu) levels were increased. Increased glutamate can be excitotoxic and cause neuronal death. As-mediated disruption of cholinergic, GABAergic, and glutamatergic systems can lead to alteration in memory consolidation and retrieval [117,118]. These mechanisms could also lead to neuronal loss in the neurotransmission pathways, and thereby cause cognitive deficits. 

### 4.2. Neurodevelopmental Defects and the Effects of Aging

It has been shown that chronic exposure to arsenic may cause detrimental neurobehavioral effects in the juvenile stage. Thus, consumption of arsenic unknowingly in childhood may have detrimental effects in later stages of life [119]. Neuropathy and peripheral neuropathy are common complications seen with arsenic toxicity. Neuropathy is a condition in which sensory function is impacted upon chronic exposure to toxicants or metabolic disorders [120]. A study in Mexico observed that urinary arsenic concentration was conversely correlated with oral IQ (verbal intellectual ability) and memory. Long term memory, attention, and capability to understand speech may be influenced by chronic exposure to arsenic in individuals with chronic malnutrition. Further, IQ of children can be decreased with increased arsenic exposure [121,122]. Additionally, by evaluating the impacts of arsenic at various life stages, studies found that early life stage exposure could lead to health impacts in adults. Clinical observations are not very well understood and poor diagnosis may lead to later complications, although as per current literature, impacts on adult life stages are much more studied than early life or prenatal exposure impacts [123].

### 4.3. Neurobehavioral Effects of Aging in Animal Models

Spatial learning ability of arsenic-exposed rats revealed impairment of spatial memory as concluded by inferior performance on hidden platform acquisition tests [124]. Another study of prenatal arsenic exposure before 4 months of age showed that the arsenic-exposed rats had increased errors in sensory information, and arsenic-exposed offspring had deficits in spatial working memory and reactivity to novelty [125,126]. A dose-dependent decline in body weight and brain weight were the result of arsenic exposure in young animals [127]. Another study reported that the levels of dopamine and serotonin in the brain increased with a decrease in norepinephrine following exposure to arsenic [112]. 

### 4.4. Neurobehavioral Effects of Aging in Humans

In infants, calamitous consequences have been associated with acute as well as chronic exposure. The outcomes of toxicity in children have been explained in a meta-analysis report. The meta-analysis performed in China showed that the mean IQ score of infants exposed to arsenic was six points less than that of unexposed infants [128]. The study also reported that the impact of arsenic toxicity depended on acute and chronic exposures. Moreover, upon aging the effects were largely mediated via chronic exposure. This suggests that low levels of arsenic in the early developmental stages, if neglected, might lead to severe complications with increasing of age [73]. Another clinical study on females aged 6 months demonstrated strong effects of arsenic on the neurodevelopment [129]. Further, emerging studies have shown that children up to 5 years of age are more prone to intellectual deficits due to arsenic exposure [74,130,131]. Neurobehavioral effects were also observed on chronic arsenic exposure in adolescents [119]. Adults who were exposed in early stages to arsenic performed poorer in neurobehavioral subsets indicating that infantile exposure to arsenic may affect behavioral development in the later stages of life. Studies with the geriatric population showed that a low level of arsenic exposure is linked with weaker cognition, decline in visuospatial skills, decline in language skills and information processing speed, impairment in the ability to execute tasks, and diminished short term memory [132]. Children exposed to arsenic through drinking water were found to have poor performance in information processing speed, although their verbal skills were not affected much [133,134]. Studies suggest that arsenic exposure through drinking water was associated with decreased IQ scores in youngsters aged between 6–10 years [131].

## 5. Toxicological Pathways

### 5.1. Molecular Mechanisms of Arsenic-Induced Oxidative Stress

**Figure 2:**
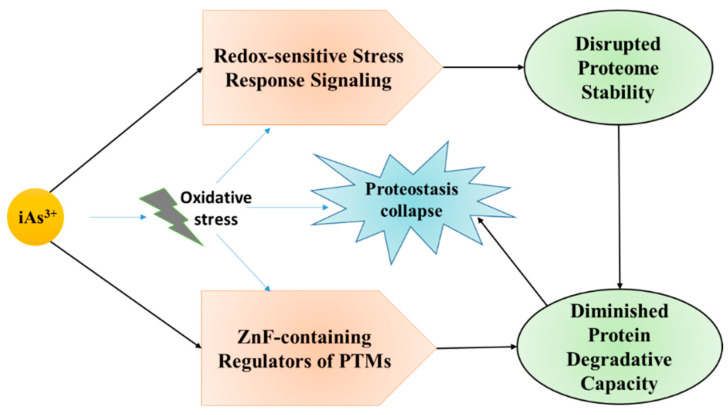
Pathway of inorganic arsenic-induced oxidative stress leading to proteostasis collapse by disrupting the functions of Zinc finger proteins (ZnF) and redox sensitive stress response signaling, which further leads to disrupted proteome stability and diminished protein degradative capacity.

Numerous in-vitro and in-vivo studies reported that arsenic toxicity (Table 1) is predominantly mediated by the induction of oxidative stress. ROS, majorly superoxide anion radical and hydrogen peroxide, were shown to increase in various tissues following exposure. Oxidative stress and inflammation are interconnected in a very complex cycle in which ROS can trigger various transcription factors that eventually upregulate the expression of prooxidative and antioxidative enzymes including the expression of proinflammatory cytokines [135]. Furthermore, phagocytic leucocytes can be activated and recruited to inflammatory sites and activate enzymes which can further potentiate oxidative stress, leading to increased inflammation. Oxidative stress and inflammation are key factors accountable for chronic diseases as shown below (Figure 2).

Several reports suggest that oxidative stress mediates arsenic-induced inflammation and neuronal dysfunction in rodent models. In a few in-vitro cell line models, arsenite was found to activate pr-inflammatory factor, NF-κB, mediated through oxidative stress [140]. Earlier studies reported increased lipid peroxidation, oxidative stress, protein carbonylation, decreased glutathione and increased glutathione disulfide and reduced antioxidant enzyme activity in various tissues [141]. When cultured microglia and astrocytes from rat hippocampus were exposed to arsenic, it was found to elevate expression of pro-inflammatory cytokines such as IL-1β, IL-6, IFNγ, and TNFα. Increased levels of these inflammatory markers mediate neuronal toxicity. Chronic exposure to arsenic also stimulates inducible NOS in various brain regions leading to nitrosative stress [142,143,144,145].

One of the primary sites for arsenic-induced oxidative stress is the mitochondrion. Exposure to arsenic primarily causes structural alterations in mitochondrial integrity which leads to a fast deterioration of mitochondrial membrane potential [146]. Altered membrane potential may cause uncontrolled formation of ROS. Further, depleting membrane integrity activates a downstream cascade of radical species causing a decline in cellular oxidative stress defense molecules such as GSH [147]. However, complete molecular mechanisms of arsenic toxicity are not fully understood; nonetheless, several studies showed that arsenic exposure enhances the production of ROS. Current literature strongly suggests that induction of ROS is the primary mechanism of arsenic-induced toxicity [148,149].

Below is a list of various mechanisms by which arsenic is believed to induce cellular oxidative stress:

(i.) Arsenic is known to induce changes in mitochondria, the mitochondrial membrane integrity and reduce membrane potential. These morphological alterations are primary sites for the unregulated production of superoxide anion radicals causing a cascade of downstream processes resulting in the formation of free radicals. The further build-up of oxidative stress leads to failure of the oxidative defense system and results in toxic manifestations [150].
(ii.) Mitochondrial complexes I and III produce O^2−^ in the electron transport chain. Arsenic inhibits succinic dehydrogenase activity and promotes uncoupling of oxidative phosphorylation with the output of O^2−^, which leads to a buildup of oxidative stress [151].
(iii.) Arsenic may also generate ROS through NAD(P)H oxidase assisted processes. NAD(P)H oxidase is a membrane-bound enzyme that produces superoxides by transferring electrons from NAD(P)H within the cell around the membrane and combining those to molecular oxygen to generate superoxide anions. It was demonstrated in mammalian endothelial cell culture that arsenic acts as an extracellular signal for the Ras proteins (cdc42), which activate NAD(P)H oxidase to generate ROS [96].
(iv.) Arsenic can also generate ROS by affecting nitric oxide (NO) synthase enzyme system. Nitric oxide synthase iso-enzymes are coupled to produce NO from L-arginine and molecular oxygen without producing superoxides. Exposure to arsenic disrupts this coupling produces ROS [97].
(v.) Metabolism of As(III) to As(V) in normal conditions results in the generation of H_2_O_2_ [98].
(vi.) ROS are generated during the formation of intermediate arsine species such as dimethylarsenic peroxyl radicals-metabolic by-products of dimethylarsinic acid [99].
(vii.) Methylated 3+ organic arsenicals react with sulfhydryl groups (-SH) in antioxidative proteins and inhibit their activity, which results in a build-up of oxidative stress [100].

### 5.2. Mitochondrial Dysfunctions

A complex relationship occurs between mitochondria and functioning of other cellular machinery affecting cell survival. Mitochondria are the powerhouses of the cell and have important roles in oxidative phosphorylation and the electron transport chain. They are also an additional major source of cellular free radicals [152]. When organisms are exposed to xenobiotics such as arsenic, they cause disruption of mitochondrial function as illustrated by inhibition of mitochondrial oxygen utilization, leading to disturbed membrane potential. Decreases in ATP levels and the membrane potential cause a disparity in the energy consumption and expenses [153].

Several previous studies have reported that arsenic-induced neurotoxicity is primarily mediated through mitochondrial dysfunction and oxidative stress. In neuropathological experiments, substantial attempts were made to investigate various molecular mechanisms in mitochondrial dysfunction caused by arsenic [154]. Data indicate that arsenic can specifically damage respiratory cycles in mitochondria, further leading to ROS production in various cells including neurons [155]. Arsenic inhibits mitochondrial complex I, II and IV activities in the brain after 12 weeks of exposure to arsenic. Arsenate can significantly impact ATP production by competing with phosphate due to its structural similarities, also known as arsenolysis, which occurs during the glycolytic pathway. Normally, in the glycolytic pathway, 1,3-biphospho-D-glycerate is formed by enzymatic linking of phosphate to D-glyceraldehyde-3-phosphate. However, in the presence of arsenate, phosphate is replaced with arsenate which leads to the formation anhydride 1-arsenato-3-phospho-D-glycerate instead of traditional 1,3-biphospho-D-glycerate. The formed anhydride is not very stable and further hydrolyzes to arsenate and 3-phospho-D-glycerate because the As-O bond length is slightly (10%) higher than P-O bond, which makes it unstable. These steps cause depletion of ATP production. ATP generation during glycolysis occurs in presence of phosphate, but in the presence of arsenate this is impacted significantly. Similarly, at the mitochondrial level, arsenolysis impacts ATP production at the oxidative phosphorylation step. At the submitochondrial level in the presence of succinate, Adenosine-5-diphosphate–arsenate is synthesized by utilizing adenosine-5-diphosphate (ADP) and arsenate. Due to structural similarities with phosphate, in the presence of arsenate instead of ATP, ADP–arsenate is produced. Unlike ATP, ADP–arsenate is not stable and is further hydrolyzed causing significant depletion of ATP [156,157,158,159].

Mice exposed subchronically to a low level of arsenic trioxide, had inhibited SDH (succinate dehydrogenase) activity and down-regulation of genes responsible for mitochondrial complex (II, IV and V) in neuronal cells. These mitochondrial complexes are encoded by mitochondrial and nuclear DNA. Further studies are required to characterize molecular pathways involved in altered gene expression on arsenic exposure [157,159,160]. Cellular metabolism and calcium have key roles in activating the pathways leading to mitochondrial membrane dysfunction. These two factors could provide further insights into the changes of membrane integrity, lipid composition, abnormalities in cytoskeleton and production of free radical ions. Limited scientific evidence for neurotoxic potential suggests a linkage between mitochondrial dysfunction and intracellular calcium levels. Arsenic-mediated destruction of mitochondrial function may cause interrupted Ca^2+^ homeostasis by altering endoplasmic reticulum buffering capability [136,137,138,161,162].

### 5.3. Demyelination and Myelination

**Figure 3:**
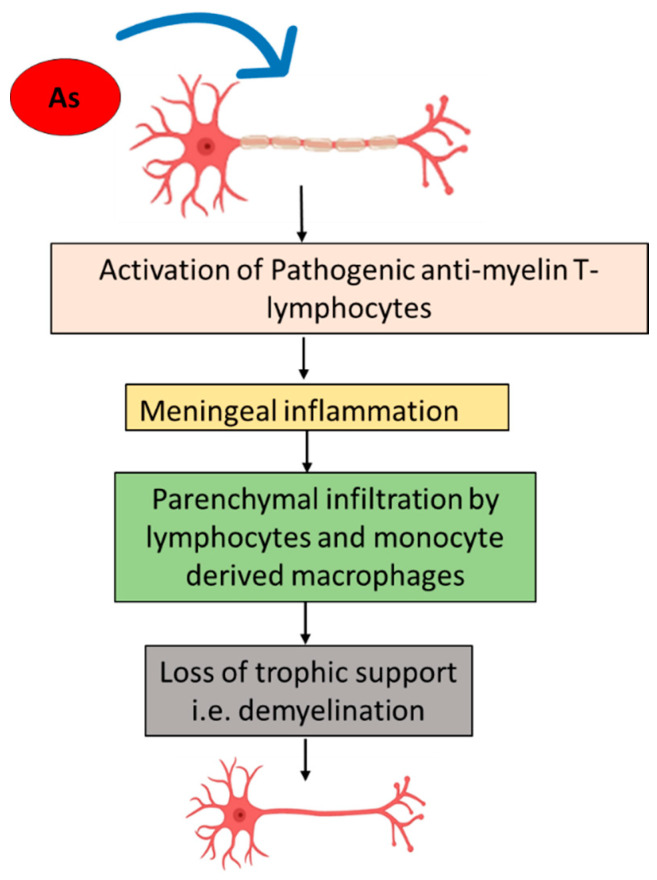
Demyelination upon arsenic (As) exposure through activation of pathogenic antimyelin T-lymphocytes leading to meningeal inflammation and parenchymal infiltration by lymphocyte and monocyte derived macrophages resulting in the loss of trophic support to the neurons.

Myelination plays an important role in the structural and functional maturation of the brain throughout life [139,163]. It is an important factor for brain plasticity and sensitive to several environmental factors, particularly in early stages of life [164]. Abnormal myelination or loss of neuronal myelin cause severe loss of brain functions including motor and sensory dysfunction [165]. Several studies concluded that environmental toxicants, including arsenic, disrupt normal brain plasticity and cause impairment in cognitive function, although the arsenic effects were inconclusive on neuronal myelination [166]. Similar observations strongly supported the above findings that loss of synaptic plasticity, learning and demyelination result from arsenic exposure [78]. At a 24 h exposure level, arsenic was shown to impairs axonal transport and the expression of neurofilaments in cell cultures by disrupting the energy generation pathway, which triggered degenerative processes leading to axonal damage and transection [167,168]. Nino et al. (2018) showed amyloid precursor protein (APP) accumulation in arsenic-exposed rats, which indicated impaired axonal transport and oxidative damage in neural membranes [83]. Hence it is evident that axons as well as myelin are the targets for arsenic exposure. Rumbeiha et al. (2014) reported that exposure to organic arsenicals (1000 ppm for 3–10 days or 250 ppm for 20–40 days) in swine through diet caused demyelination of peripheral nerve fibers, ataxia and paralysis of hindquarters [169]. A recent study reported demyelination of the cerebral cortex and callosum is linked with arsenic exposure. In response to this, compensatory mechanisms such as mitochondrial adaptive/stress responses are activated [170]. Donofrio et al., (1987) [171] reported that four patients with subacute polyradiculoneuropathy following arsenic poisoning experienced segmental demyelinating polyradiculoneuropathy involving both humoral and cell-mediated immune mechanisms. This study also showed that acute arsenic intoxication may cause Guillain-Barre syndrome. Acute inflammatory demyelinating polyradiculoneuropathy (AIDP) is characterized by an immune-mediated attack on myelin with infiltration of macrophages and lymphocytes with segmental stripping of myelin as shown in Figure 3.

### 5.4. Effects on Nerve Conduction

**Figure 4:**
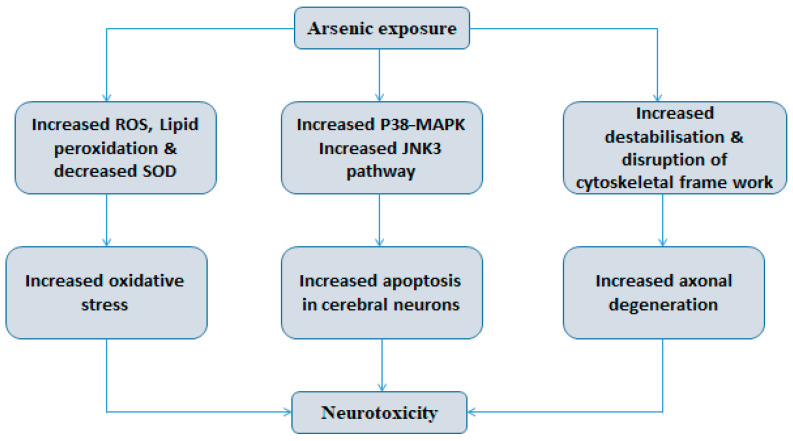
Three pathways of arsenic-induced neurotoxicity: (i) increase in ROS activity, lipid peroxidase and decrease in SOD activity leading to increased oxidative stress causing neurotoxicity; (ii) increased P38-MAPK, JNK3 pathway leading to increased apoptosis in cerebral neurons causing neurotoxicity; (iii) Increased destabilization, disruption of cytoskeletal framework and axonal degeneration.

There are several pathways described for arsenic-induced neurotoxicity (Figure 4). Motor and sensory nerve conduction measures how fast an electrical impulse moves through a nerve. During this nerve conduction velocity test, the particular nerve is stimulated with electrode patches attached to the skin. This test is used to measure nerve damage and dysfunction. Tseng et al. reported that chronic arsenic exposure led to slow nerve conduction velocity in adolescents in Taiwan, and it was reported that a decrease in nerve conduction velocity of the sural saphenous nerve (SAP), which supplies sensation to the skin, can be used as an early marker for chronic arsenic neuropathy [172]. In this study, people who were chronically exposed to arsenic showed a marked decrease in nerve conduction (including the median saphenous nerve, ulnar compound muscle action potential, the ulnar saphenous nerve and the sural saphenous nerve). Taller subjects (>163 cm) were reported to have decreased nerve conduction velocity than shorter subjects. Several studies have reported that in symmetrical peripheral neuropathy, sensory nerves are more sensitized than motor nerves, and the larger arm neurons are greatly affected [173]. Arsenic exposure caused fragmentation and resorption of myelin on the distal portion of nerves, with disintegration of axis cylinders and reduction in the number of myelin fibers. Several other studies reported encephalopathy, and impairments of superior neurological functions are linked to arsenic exposure [174,175].

### 5.5. Overview of Epigenetical and Transgenerational Effects

Epigenetics is the research of factors influencing heritable changes without alterations in the DNA sequence (phenotypic change without genotype change). Epigenetics is a highly regulated mechanism that controls the expression of genes during the developmental phase of an organism. Epigenetic mechanisms restructure chromatin and regulate the accessibility of DNA to transcriptional factors based on physiological conditions, which eventually leads to higher or lower expression of genes. Three different mechanisms, including histone modifications, noncoding RNAs (ncRNA) and DNA methylation are responsible for gene silencing, and are believed to initiate and sustain epigenetic changes. Epigenetic modifications are influenced by several factors such as age, lifestyle, environment, exposure to pollutants, and pathological conditions of the organism [176]. 

Metabolism of arsenic consumes a significant amount of S-adenosylmethionine (SAM), a primary methyl donor molecule in DNA methylation, which is a process mediated by DNA methyl transferases (DNMTs) [176]. Abnormal DNA methylation patterns have been reported in zebrafish embryos after acute exposure to arsenic [94]. Such abnormalities in DNA methylation might take place due to the impacts of arsenic on SAM-dependent methyltransferases, eventually changing histone and DNA methylation patterns [177]. Mirbahai et al., (2013) reported variation of global DNA methylation and disruption of one-carbon metabolism, causing hepatic carcinogenicity in fish from As-contaminated aquatic systems. These effects were linked to a decrease in choline and DNA methyltransferases activity through elevation of the DNA methyltransferases inhibitor S-adenosyl homocysteine [178]. 

Post-translational alteration of histones restricts gene expression by modifications in chromatin structure and, consequently, accessibility of DNA to transcription machinery. Histone methylation inhibits gene expression, whereas demethylation triggers gene expression. Furthermore, histone acetylation increases gene expression by making genes accessible for transcription machinery. Arsenic alters pyruvate dehydrogenase (PDH) activity, which is predominantly responsible for the formation of acetyl-CoA through the oxidation of pyruvate [179]. Acetyl-CoA is a crucial substrate for acetylation, thus arsenic exposure will significantly impact histone acetylation. Prenatal exposure to arsenic in mice causes hypo-acetylation at H3K9, which results in impaired episodic and spatial memory. In contrast, developmental exposure to arsenic can cause gender-specific regulation of H3K9 acetylation and methylation [180].

Current literature suggests that arsenic can cause epigenetic alterations by different mechanisms and inhibiting the activity of DNMTs is one of the most notable among them. DNMTs are responsible for catalyzing methyl group transfer from SAM onto the C5′ position of cytosine at CpG dinucleotide of the promoter region of genes to produce 5-methylcytosine, which leads to hypermethylation of promoter regions and subsequent gene silencing. Evidence also suggests that promoter demethylation, along with an inhibition of DNMTs 1, 3a, and 3b, can cause altered gene expression. However, inhibition of DNMT activity can lead to detrimental impacts on animals. Human epidemiological studies confirm that exposure to arsenic via drinking water and food correlates significantly with DNA hyper methylation. High arsenic levels in drinking water in various regions of the world have been linked to global DNA hypomethylation accompanied by epigenetic silencing of some critical genes, increasing the risks of cancer [176]. In addition, experimental findings suggest that targeting epigenetic modifications might be a fruitful approach to combat arsenic toxicity [181].

Recently, Valles et al. (2020) demonstrated the possible transgenerational effects of arsenic in zebra fish. In this study, F_0_ embryos were directly exposed to waterborne arsenic (50 and 500 ppb) for 150 days. Arsenic exposure to F_0_ generations altered motor activity during the development phase and increased anxiety-like behaviors, which were transmitted to the F_2_ generation. The most interesting observation was the reduction of brain-derived neurotropic factor (BDNF) expression at mRNA levels in F_0_ and F_2_ adults, and increase histone H3K4me3 methylation. Hypermethylation of the BDNF gene promoter region observed in F_0_ and F_2_ generations was likely the reason for decreased expression levels of the BDNF gene. BDNF is crucial for neurodevelopment, including synaptic plasticity and regeneration, which are essential for cognition and memory. Changes in BDNF levels could also lead to anxiety-like disorders. When behavior tests were performed in the F_2_ generation of the above-mentioned study, fish demonstrated spontaneous tail coiling and anxiety-like behavior, and a decrease in larval and adult motor activity and exploratory behavior [115]. This indicates that chronic exposure to arsenic can cause trans-generational effects in zebra fish by epigenetic alterations.

### 5.6. Proteinopathy and Arsenic Toxicity

**Figure 5:**
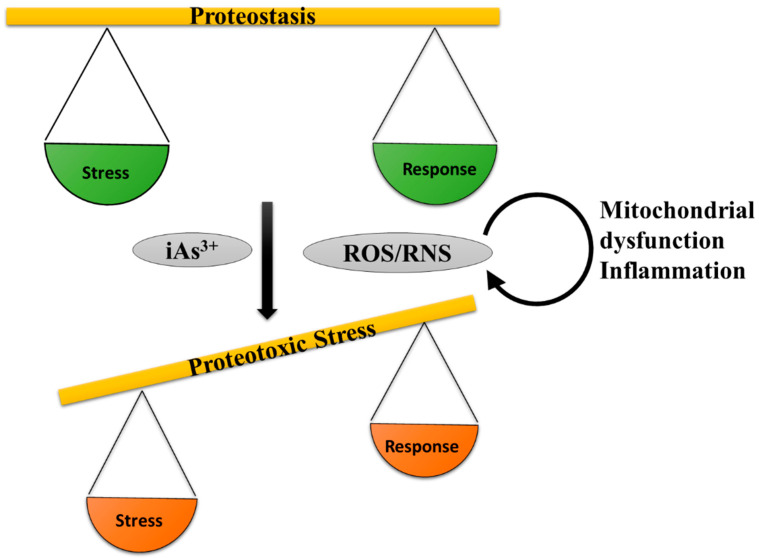
Arsenic-elicited over-production of ROS/RNS alter the equilibrium of proteostasis.

Proteinopathy is usually a term for diseases which are characterized by the production of divergent conformers of a certain protein that are misfolded and aggregated, which leads to dysfunctions. As a result of aging, the mechanism responsible for protein synthesis is deregulated in the central nervous system (CNS). It has been reported that a low level of arsenic exposure can upregulate the expression of APP, which, in turn, induces the levels of Aβ, although it is still unknown whether this behavioral deficit is associated with proteinopathy [182,183]. Molecular chaperones are a group of proteins that have functional similarity and modulate the proteostasis network by sensing and proofreading misfolded peptides to avoid aggregation [184]. It has been suggested that exposure to arsenic leads to the disruption of the functions of molecular chaperones [185]. As a division of the cellular stress response, inorganic arsenic (As^3+^) was found to induce HSP70 and small HSP’s expression. Furthermore, chronic arsenite exposure was shown to cause overexpression of heat-shock proteins via activation of DAF-16/FOXO transcription factor [186,187,188]. Arsenic exposure may also alter protein clearance by disrupting the ubiquitin-proteasome system (UPS) and axonal transport of the damaged proteins. Thus, chronic arsenic exposure results in a progressive decrease in the capacity of protein refolding and clearance of aggregated proteins; causing protein misfolding as depicted in Figure 5.

## 6. Strategies for the Removal of Arsenic

Research has improved several treatment strategies for the removal of arsenic from water, the community and household premises. Removal of pentavalent arsenic is more effective compared to other forms. A two-step method is being approached to treat arsenic contaminated, which includes pretreatment in which oxidation of As(III) to As(V) occurs followed by a targeted method [189]. Conventional methods for removal of arsenic from water involve adsorption, oxidation, precipitation, coagulation-flocculation, ion exchange, electrocoagulation, membrane technologies, electrochemical arsenic remediation, phytoremediation and microbial remediation. Oxidation is considered to be a prerequisite process which converts As(III) into As(V), which is a more stable form; removal of this As(V) takes place by adsorption/sedimentation/filtration or other techniques. Pettine et al. [190] demonstrated that oxidation of As(III) by H_2_O_2_ is favorable in the presence of Fe^2+^ and Cu^2+^ as these cations exert a catalytic effect on oxidation of As(III). Moving to the coagulation flocculation process, this was shown to be effective in the removal of arsenic from water. It is a pH sensitive process, so whenever the pH is greater than 7.5 ferric chloride has better performance for the removal of As(V) [191,192]. The presence of silicate and phosphate affects the performance of the coagulation process [193]. Electrocoagulation is an emerging technique that has been applied in the treatment of urban, potable and waste water with heavy metals, and colored water [194,195]. Yilmaz et al. [196] reported that this process has three times higher effectivity for removal of the contaminants. Among all these treatment processes, adsorption may be widely used and is an economically feasible method. Many studies reported that the adsorption process has shown effective performance towards the removal of odor, color and different organic and inorganic contaminants from the water [197,198,199,200]. Different types of adsorbents such as activated alumina, activated carbon, iron-based adsorbents and other miscellaneous adsorbents are used [201,202,203,204]. Ion exchange is a physicochemical process in which ions are exchanged between resins and feed water. This process is considered promising in the removal of arsenic. It has been reported that by using ion exchange process it is possible to bring the effluent concentration of arsenic to below 10 µg/L [205]. For field-based arsenic removal techniques such as flocculent-disinfectant [206], hybrid ion exchange [207], solar oxidation [208], a bucket treatment unit [209], and adsorption using activated laterite [210] were developed to provide arsenic-free water at the household level. Life cycle assessment is a tool for evaluating the sustainability of water treatment by considering all technical, environmental, social and economic aspects [211]. Accordingly, abundance of arsenic in groundwater, soil, crops and ores is required to meet the urgency and demands of public health. However, as arsenic is rapidly oxidized from As(III) to As(V), the nature of the toxic profile and risk also get changed with forms. It is imperative to remove/target As(III) due to its hazardous effects on human health. Several treatment parameters have been considered to assess how life cycle assessment can be used as an effective tool to provide a reliable assessment of the sustainability of targeted treatment systems.

## 7. Conclusions

Arsenic exposure affects millions of people globally, and epidemiological evidence provides an imperative guide to arsenic risk assessment in food, water and air. Understanding the neurotoxic impacts of arsenic is crucial, since exposure is a global public health concern and arsenic levels in the environment are rising every year around the world due to increased anthropogenic activity. The epidemiological evidence undeniably indicate that arsenic significantly affects cognitive functions and intellectual ability during development. Although several toxicological and epidemiological reports have demonstrated neurological impacts of arsenic, the underlying mechanisms for such effects are poorly understood and need to be explored further. Nonetheless, arsenic appears to be a strong neurotoxicant that elicits its effects, essentially, by inducing oxidative stress in the brain [212].

In this review, we summarized our current understanding of the various molecular mechanisms of neurotoxicity. The available literature suggests that there is a need to focus on arsenic-induced neurotoxicity mechanisms. However, animal studies raise the prospect of oxidative stress-mediated mitochondrial dysfunction in inducing neurotoxicity. The deeper exploration of arsenic-induced oxidative stress and mitochondrial dysfunction may help to devise therapies for alleviating arsenic-induced neurotoxicity. A detailed investigation of the epigenetic signatures of arsenic and its impacts on metabolism is needed, as this has huge capacity to explore biomarkers for disease progression. Additionally, the effect of cumulative exposures to arsenic and its progression to neurotoxicity has been poorly studied. Arsenic induced ROS generation and subsequent lessening of cellular antioxidant defenses can result in disorder of redox equilibrium in tissues. Because of its sulfhydryl group binding capacity, arsenic has been shown to alter the activities of various cellular enzymes, specifically those which play major roles in controlling fatty acid oxidation and glutathione production. However, more elaborative and decisive research is required to characterize the molecular pathways that link oxidative stress and epigenetic mechanisms leading to the transgenerational impacts of arsenic. Although several studies have focused on these aspects, an in-depth understanding remains elusive.

## Figures and Tables

**Table 1:** In-vitro and in-vivo studies demonstrating mechanisms of arsenic neurotoxicity.

Arsenic	Species	Exposure Duration	Pathological Mechanisms	Toxic Outcome	Ref.
Sodium arsenite	Rat	4 months	Increased APP (amyloid precursor protein) and RAGE 9, also increased enzymatic activity of BACE1 (β-secretase).	Neurodegeneration disorders associated with amyloid accumulation.	[2]
Sodium arsenite	Rat	1 month	Increased lipid peroxidation and decrease in nerve conduction velocity. myelin thickness, area, and perimeter of axons.	Impaired central nervous system.	[3]
Sodium arsenite	Rat	9 h	Absence of neurofilament and fibroblast Proteins.	Altered cytoskeletal composition	[4]
Sodium arsenite	Rat	28-days	Reduction in superoxide dismutase-2 and Catalase action in hippocampus, striatum and cortex.	Altered locomotor activity and grip strength.	[5]
Sodium arsenite	Rat	4 months	Elevated oxidative stress, lipid peroxidation and reduced glutathione levels in brain mitochondria.	Increased oxidative stress and mitochondrial damage.	[39]
Sodium arsenite	Rat	28-days	Increased oxidative stress. Decrease in superoxide dismutase-2 activity.	Increased apoptosis in brain cells.	[7]
Sodium arsenite	Rat	10-weeks	Decrease in antioxidative defense mechanisms (GPx, GST, MnSOD, CAT and GR), enhanced LPO observed in the mitochondria at cerebral cortex, cerebellum and hippocampus.	Significant impact on behavioral functions like total locomotor activity, open field behavior, exploratory behavior and grip strength.	[136]
Sodium arsenite	Rat	28-days	Increased oxidative stress in frontal cortex and hippocampus. Increased levels of Nrf2 and HO-1 proteins.	Demise of myelin sheath in neurons and imprecise cristae in the mitochondria both hippocampal and frontal cortex regions. Cholinergic deficits detected.	[137]
Sodium arsenite	Rat	3 months	Biochemical and molecular modifications via inducing oxidative stress and dysfunction of mitochondria.	Mitochondrial decreasing complexes activity and functional impairment.	[31]
Sodium arsenite	Rat	3 months	Reduced NR2A expression in the hippocampus.	Impaired memory.	[10]
Sodium arsenite	Rat	3 months	mGluR5 mRNA and protein expression in hippocampus and cortex.	Learning and memory ability declined.	[11]
Sodium arsenite	Rat	30 days	Lowered expression of NMDAR NR2B subunit and EAAC1 in the brain (hippocampus).	Spatial memory impairment.	[12]
Arsenic trioxide	Mice	45 days	Significant raise in lipid peroxidation, glycogen in cerebral hemisphere and cerebellum.	Neurotoxic effects.	[13]
Arsenic trioxide	Mice	60 days	Reduction of Sdha expression and activity in brain, mitochondrial respiratory chain genes downregulation.	Neurodegeneration disorders.	[14]
Arsenic trioxide	T98G and A172 cells	6, 8 and 24 h	Aggregated mitochondria and MMP dissipation.	Induced apoptosis.	[138]
Arsenic trioxide	SY-5Y cells	24, 48 and 72 h	Elevated intracellular calcium ions.	Increased occurrence of apoptosis and DNA damage.	[139]
Sodium arsenite	Primary astrocytes	24 h	Decreased mitochondrial membrane permeability and decreased protein expression of GLT-1, GS, and GLAST.	Inhibit glutamate metabolism leading to neurotoxicity.	[67]
Arsenic trioxide	Rat neuronal cells	8 h	Increased expression of calpain 1, cdk5, p25 levels.	Induced neuronal cell apoptosis.	[69]
Arsenic trioxide	Neuro-2a cells	24 h	Oxidative stress damage decreased Nrf2 and thioredoxin expression. Mitochondrial dysfunction, PARP activation and caspase cascades, caspase-3 activity.	Neuronal cell death.	[70]
Sodium arsenite	Bergmann glial cells	24 h	Increased EAAT1/GLAST activity and decrease in GLU transport.	Neuronal damage.	[71]

## Data Availability

Not applicable.

## Abbreviations

AD	Alzheimer’s disease
APP	Amyloid precursor protein
As	Arsenic
BDNF	Brain-derived neurotrophic factor
CRFR1	Corticotrophin-releasing factor
DA	Dopamine
EPA	Environmental protection agency
GSH	Glutathione
HPA	Hypothalamic-pituitary-adrenal
MMA	Monomethyl arsonate
NCS	Nerve conduction studies
NCV	Nerve conduction velocity
NO	Nitric oxide
PDH	Pyruvate dehydrogenase
ROS	Reactive oxygen species
RBC	Red blood cells
SOD1	Superoxide dismutase
SOD2	Superoxide dismutase
SDH	Succinate dehydrogenase
SAM	S-adenosylmethionine
-SH	Thiol
UPS	Ubiquitin-proteasome system
WBC	White blood cells
WHO	World health organization
